# Polio Endgame: Lessons Learned From the Immunization Systems Management Group

**DOI:** 10.1093/infdis/jiw592

**Published:** 2017-07-01

**Authors:** Simona Zipursky, Jos Vandelaer, Alan Brooks, Vance Dietz, Tasleem Kachra, Margaret Farrell, Ann Ottosen, John L. Sever, Michel J. Zaffran

**Affiliations:** 1 World Health Organization, Geneva, Switzerland;; 2 UNICEF Programme Division, New York, New York;; 3 Gavi, the Vaccine Alliance, Geneva, Switzerland;; 4 US Centers for Disease Control and Prevention, Atlanta, Georgia;; 5 Bill & Melinda Gates Foundation, Seattle, Washington;; 6 Rotary International, Evanston, Illinois; and; 7 UNICEF Supply Division, Copenhagen, Denmark

**Keywords:** polio, polio eradication, endgame, routine immunization, vaccines, IPV, OPV, inactivated polio vaccine, oral polio vaccine.

## Abstract

The Immunization Systems Management Group (IMG) was established to coordinate and oversee objective 2 of the Polio Eradication and Endgame Strategic Plan 2013–2018, namely, (1) introduction of ≥1 dose of inactivated poliovirus vaccine in all 126 countries using oral poliovirus vaccine (OPV) only as of 2012, (2) full withdrawal of OPV, starting with the withdrawal of its type 2 component, and (3) using polio assets to strengthen immunization systems in 10 priority countries. The IMG’s inclusive, transparent, and partnership-focused approach proved an effective means of leveraging the comparative and complementary strengths of each IMG member agency. This article outlines 10 key factors behind the IMG’s success, providing a potential set of guiding principles for the establishment and implementation of other interagency collaborations and initiatives beyond the polio sphere.

The Polio Eradication and Endgame Strategic Plan 2013–2018 (the Endgame) [1], calls for the global public health community to unite to achieve four critical objectives: (1) poliovirus detection and interruption, (2) immunization program strengthening and oral poliovirus vaccine (OPV) withdrawal, (3) containment and certification, and (4) legacy planning. To meet these objectives, the Global Polio Eradication Initiative (GPEI) established a series of management groups, each responsible for one of these objectives, or for a cross-cutting function (ie, budget, advocacy).

The Immunization Systems Management Group (IMG) was established to coordinate and oversee objective 2 of the Endgame, namely (1) introduction of ≥1 dose of inactivated poliovirus vaccine (IPV) into the routine immunization (RI) program of all 126 countries using only OPV as of 2012; (2) full withdrawal of OPV, starting with the withdrawal of its type 2 component through a transition from trivalent OPV (tOPV) to bivalent OPV (bOPV) in 155 tOPV using countries and territories; and (3) use of polio assets to strengthen immunization systems in 10 priority countries. The commitment from all 126 countries using OPV only to introduce IPV by the end of 2015, along with the smooth implementation of the switch from bOPV to tOPV (hereafter “the switch”), and the systematic integration of RI and polio activities in some of the most challenging countries are all testaments to the IMG’s success.

## ESTABLISHING THE IMG

Established in April 2013, the IMG was a unique management group from the outset. GPEI relied on its staff focused on poliovirus surveillance, outbreak response, advocacy, and finance to lead its management groups. In contrast, GPEI delegated responsibility for the implementation of objective 2 to existing staff working on RI within each of GPEI’s partner agencies. This decision was strategic. Not only were the polio staff fully occupied with polio campaigns and outbreak response (ie, objective 1), but there also was a benefit in incorporating staff with experience with the Expanded Programme on Immunization (EPI), strengthening of immunization systems, and introduction of new vaccines into the RI programs.

The IMG was cochaired by a senior staff member from the EPI team of the World Health Organization (WHO) headquarters and another from the United Nations Children’s Fund’s (UNICEF) Program Division’s Immunization team. The IMG core comprised two members from each GPEI core partner agency—the Bill & Melinda Gates Foundation, Rotary International, US Centers for Disease Control and Prevention, UNICEF, and WHO—a lead representative from the immunization team, a representative from the polio team. The IMG core members also included a representative from UNICEF’s Supply Division, the agency responsible for vaccine procurement for the GPEI. 

Given the critical task of IPV introductions in 126 countries over a short period of time required under objective 2, the cochairs decided early on that, rather than developing a parallel process for IPV introduction, they should collaborate with Gavi, the Vaccine Alliance (hereafter Gavi) and use its existing channels for support of new vaccine introduction. With the endorsement of GPEI and the Gavi board, it was agreed that for the 73 low- and lower-middle-income countries that either were eligible for Gavi support or had recently graduated from such eligibility, support for IPV introduction would be channeled through Gavi. Furthermore, given Gavi’s extensive role in both IPV introduction and support for strengthening RI, the IMG secured the permission of GPEI leadership to include the Gavi Secretariat as members of the IMG.

One of the first tasks facing the IMG was to clarify its mandate and define its responsibilities in order to focus its efforts and avoid duplicating the work of the other GPEI management groups. The IMG set out five objectives for its work: (1) clear recognition and understanding of the rationale for and urgency of the Endgame, in particular the objective 2 activities; (2) ensuring the availability of the necessary vaccines—IPV and bOPV; (3) introducing IPV; (4) withdrawing tOPV in 2016 and replacing it with bOPV (the switch); and (5) using GPEI resources to help strengthen RI in 10 focus countries (Afghanistan, Angola, Chad, Democratic Republic of the Congo, Ethiopia, India, Nigeria, Pakistan, Somalia, and South Sudan).

The IMG’s reporting lines were to the WHO’s Strategic Advisory Group of Experts on Immunization (SAGE), which advises the Director-General of WHO on immunization-related issues, and to the Polio Oversight Board (POB), responsible for overseeing the Endgame implementation, reporting through GPEI’s Strategy Committee, which has the overall responsibility of coordinating and tracking the implementation of the Endgame. This dual reporting line ensured that there was engagement from both the RI and polio decision-making bodies in implementing objective 2.

**Figure 1.**
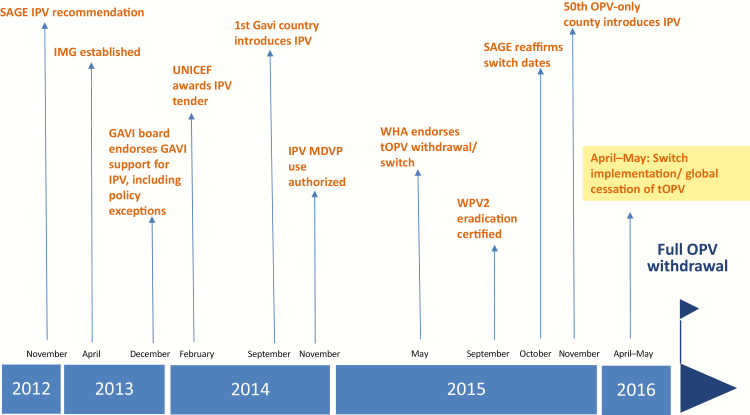
Immunization Systems Management Group (IMG) milestones. Abbreviations: IPV, inactivated poliovirus vaccine; MDVP, multidose vial policy; OPV, oral poliovirus vaccine; SAGE, Strategic Advisory Group of Experts on Immunization; tOPV, trivalent OPV; UNICEF, United Nations Children’s Fund; WHA, World Health Assembly; WPV2, wild poliovirus type 2.

One key delineations made by the IMG was that its work should be focused on implementation, as the IMG was not a policy-making body (Figure 1). This meant that, for example, SAGE set the policy for IPV introduction, while the IMG worked with countries to implement the recommendation. The IMG set up five subgroups to take responsibility for the key areas of work: the implementation subgroup (which established working groups on IPV introduction, IPV supply, and the switch) and the regulatory, communications, financing, and RI strengthening subgroups. Each subgroup was made up of representatives from the IMG partner agencies.

The IMG recognized that the leadership and support of WHO and UNICEF regional staff working on IPV introduction, the switch, and RI strengthening were critical, and it brought them on board from the outset. In addition, the IMG engaged a broader range of interested partners and organizations, beyond the IMG core agencies. This approach allowed the subgroups to be established with the right blend of expertise.

Initially, the IMG met weekly by teleconference to address the many activities that needed to be launched against a tight time frame; over time this frequency was gradually reduced to monthly meetings. Face-to-face meetings were organized approximately every 6 months. Together, these meetings were a critical facet in building momentum for the work on objective 2 and in creating strong links across organizations.

With use of the Endgame’s targets and mapping out of the information needed for discussions at SAGE meetings and with the Gavi Board, a detailed work plan was established for the IMG and its subgroups and tracked monthly to measure progress and identify any potential issues. The IMG agreed to track country level progress using the WHO Immunization Repository (https://extranet.who.int/immunization_repository/dhis-web-commons/security/login.action;jsessionid=41C340498058798AF7743385F6A6194, accessed 15 September 2016), an existing online database populated by WHO, UNICEF, and other partners to track national immunization programs information. Regular reports from both of these tools allowed the IMG to monitor progress and formed the basis of the IMG’s regular updates to SAGE and the POB through the GPEI Strategy Committee.

## SUCCESS FACTORS

The IMG was an effective means of aligning and leveraging the comparative and complementary strengths of its members. The IMG’s work was significantly enhanced because of this collaborative approach. Each IMG member organization contributed toward the achievement of objective 2 in their core area of strength and gave space to the others to contribute where they were strongest. The IMG focused on coordinating efforts among member organizations and ensuring that nothing fell through the cracks.

In addition to establishing a strong collaboration, 10 factors have been identified as having played a critical role in achieving the work of the IMG: (1) clear role and goals, (2) strong global leadership, (3) transparency and inclusivity, (4) regional leadership and engagement, (5) country leadership and support, (6) prioritization using a risk-based approach, (7) flexibility and adaptability, (8) leveraging SAGE and the POB, (9) proactive communications, and (10) common tools. These factors are described in more detail below.

### Clear Role and Goals

The IMG was given a clear mandate in objective 2: introduce ≥1 dose of IPV in countries using only OPV by end of 2015, withdraw all tOPV in 2016 and introduce bOPV, and strengthen RI programs in focus countries (Afghanistan, Angola, Chad, Democratic Republic of the Congo, Ethiopia, India, Nigeria, Pakistan, Somalia, and South Sudan). These goals, with their stated (although extremely ambitious) timelines, gave the IMG’s work a clear sense of purpose as well as a sense of urgency. Close to 190 persons were involved in the work of the IMG and its subgroups, as well as thousands in countries around the world who were working on IPV introduction, the switch, and RI; ensuring that everyone was fully informed and moving in the same direction was critical. The IMG relied on the strengths of each organization to implement the activities and left the policy making to bodies such as SAGE. Its efforts focused on coordinating activities to meet a set of specific and agreed-on goals at global, regional, and country levels.

### Strong Global Leadership

Having the WHO EPI Coordinator and the Chief of Immunization at UNICEF as cochairs of the IMG gave the work legitimacy, facilitating buy-in at all levels of their organizations. In addition, their positions within their respective agencies meant they had already developed strong networks across both their own and partner organizations, on which they were able to draw to build support for and engagement in the IMG’s work and mandate. The cochairs’ relatively senior roles within their own organizations gave them direct access to the necessary policy makers and leaders within the broader immunization community, as well as the ability to mobilize their own staff to support the work.

Perhaps most critically, the cochairs worked in partnership, supported by a dedicated secretariat at WHO along with surge support from the Task Force for Global Health. The cochairs regularly put the work of the IMG above politics and found ways to use each IMG partner agency for its respective strengths, helping to mitigate each other’s weaknesses.

At global level, high-level commitment was critical to mobilizing countries; joint letters were sent to ministers of health from the Director-General of WHO, the Executive Director of UNICEF, and the Chief Executive Officer of Gavi, as appropriate, to all OPV-only countries to encourage IPV introduction within the timelines. The WHO Director-General and UNICEF Executive Director sent a second joint letter to highlight the importance of meeting switch timelines. This level of collaboration and leadership, which was matched at regional and country levels, cannot be overstated and is described in further detail below.

The time-bound nature of the Endgame served as a motivator for all those involved. Strong leadership at all levels led to the proactive identification of problems, flexibility to think out of the box to find solutions when needed, and the ability to advocate for the resources needed to implement activities under tight timelines.

### Transparency and Inclusivity

From the outset, the IMG sought to work in collaborative, open, and transparent ways. The responsibility of chairing the five IMG subgroups was divided up among the IMG partners, which helped build an inclusive team atmosphere and accountability at all levels.

Regional colleagues were invited to join all IMG meetings and subgroup calls. Discussions were summarized and circulated broadly. Interested partners such as the Clinton Health Access Initiative, Agence de Médicine Préventive, John Snow International, the Program for Appropriate Technology in Health, and WHO’s Immunization Practices Advisory Committee were invited to participate in IMG subgroups and contribute their skills and expertise in specific areas. This inclusive approach was critical, especially in the area of IPV introduction, where the timelines were extremely tight.

The IMG also engaged in regular dialogue with IPV and OPV manufacturers and the national regulatory agencies with oversight of production facilities to ensure that they too were working toward the same goals, ensuring full transparency on program objectives. This dialogue was critical to ensuring the available IPV was allocated in line with program priorities, particularly because the global IPV supply became increasingly constrained from 2014 onward owing to setbacks manufacturers encountered in scaling up production and rising demand for IPV use in mass vaccination campaigns [2].

### Regional Leadership and Engagement

The IMG recognized early on that with 126 countries involved in IPV introduction and at least 155 countries and territories that would need to be involved with tOPV withdrawal, leadership by regional colleagues, particularly from WHO and UNICEF, was critical. The IMG relied heavily on their guidance and input in developing its work plan.

Regional offices led the activities for their respective regions, with the IMG—working largely through the implementation subgroup—providing support as requested. This support took a different form for each region, based on their specific needs. Recognizing the heavy burden this work placed on regions, UNICEF and WHO regional offices were offered resources to help augment their human resource capacity. This augmentation was tailored to each region and included seconded staff recruited by the Task Force for Global Health, creation of short-term technical officer posts, and hiring of consultants. Collaboration across WHO and UNICEF regional offices was also key. The IMG established a process by which its budget for activities was divided across WHO and UNICEF, and the agencies thus worked in partnership to develop their annual plans and support countries, avoiding duplication of efforts. Regional participation in regular calls with the various IMG subgroups provided an opportunity to track regional progress, proactively identify any issues of concern, and provide support as needed.

### Country Leadership and Support

Given the extremely ambitious timelines laid out in the Endgame, and the need for a large number of countries to implement activities in a synchronized manner, the IMG recognized that active country leadership and cooperation would be critical, and that exceptional targeted financial and staffing support would need to be provided to certain countries.

The IMG began engaging immunization staff at the country level from the outset, and in particular encouraged engagement with national immunization technical advisory groups to support decision making on IPV introduction. Given the ambitious Endgame timelines, countries eligible for and graduating from Gavi support had been offered support committed by donors to the Endgame but implemented by Gavi for IPV introduction. However, the IMG recognized that for many middle-income countries. which were not eligible for Gavi funding, catalytic support would be necessary to ensure that they would be able to introduce IPV by the Endgame targets, although such middle-income countries would then take over the financing responsibility after this initial support. The IMG advocated with the POB and received approval to provide support to these countries. Likewise, to ensure that all 155 countries and territories executed the global withdrawal of OPV type 2 in a synchronized manner, the IMG recognized that financial support would need to be extended on a limited basis to these countries, which the POB approved [3].

The IMG’s commitment to meet country needs went beyond the financial sphere. The IMG’s implementation subgroup, together with WHO and UNICEF regional offices, and the support of the Task Force for Global Health, organized trainings to ensure that a cadre of trained consultants were ready to support countries as needed.

### Prioritization Using a Risk-Based Approach

To focus its efforts and resources efficiently, the IMG operated using a risk-based prioritization approach in which countries were divided into tiers based on the assessed risks for outbreaks of polio caused by type 2 circulating vaccine-derived polioviruses they would face once OPV type 2 was withdrawn (Table 1) [3]. All IMG partners as well as SAGE endorsed this approach and the tier criteria. The IMG’s supply working group used this risk-based approach to allocate IPV, and the IMG itself used it to prioritize technical assistance when needed, as well as to identify countries for financial support.

**Table 1.** Summary Definitions of Risk Tiers for IPV Introduction Based on Risk of cVDPV2 Outbreaks and Importations After Cessation of the Type 2 Component of OPV

Tier	Definition
Tier 1	WPV-endemic countries *or* countries that have reported a cVDPV2 since 2000
Tier 2	Countries that have reported a cVDPV1/cVDPV3 since 2000 *or* large/medium-sized^a^ countries with DTP3 vaccine coverage of <80% in 2012, 2013, or 2014, according to WUENIC
Tier 3	Large/medium-sized^a^ countries adjacent to tier 1 countries that reported WPV since 2003 *or* countries that have experienced a WPV importation since 2011
Tier 4	All other countries using OPV

Abbreviations: cVDPV1, cVDPV2, and cVDPV3, circulating vaccine-derived poliovirus types 1, 2, and 3, respectively; DTP3, third dose of the diphtheria-tetanus-pertussis vaccine; IPV, inactivated poliovirus vaccine; OPV, oral poliovirus vaccine; WPV, wild poliovirus; WUENIC, World Health Organization/United Nations Children’s Fund Estimates of National Immunization Coverage.

^a^Small, medium-sized, and large countries were defined as those with <20 000, 20000 to 1 million, or >1 million live births, respectively.

### Flexibility and Adaptability

Key to the IMG’s success was its willingness to be flexible and adapt to consistently changing situations. For example, the Gavi Secretariat leveraged its existing business model, which GPEI donors were already comfortable with, to help encourage rapid release of funds for IPV procurement and technical assistance. In addition, the Gavi Secretariat developed a new expedited process for countries to apply for IPV introduction support, which included the waiving of its standard requirements, such as cofinancing and minimum coverage thresholds. WHO organized regional workshops of national regulatory authorities to streamline the licensing of IPV and bOPV and prioritized the review of prequalification dossiers to ensure that vaccine supply could be available as soon as possible. 

When it became clear in mid-2014 that the available IPV supply would be insufficient to meet demand, WHO convened special scientific committees and launched studies to explore the feasibility of safely implementing measures to stretch existing supply (eg, increasing the length of time that an open vial of IPV could be safely used, fractional dosing of IPV delivered intradermally). At the Centers for Disease Control and Prevention, funds and personnel were made available on short notice to conduct several studies regarding health care providers’ and child caregivers’ acceptance of the administration of multiple injectable vaccines at a single visit [5], a key concern around adding IPV to the RI schedules in many countries. At UNICEF, new positions were fast-tracked for creation at regional levels to ensure adequate support for IPV introduction. The Bill & Melinda Gates Foundation secured the involvement of the Task Force for Global Health to provide surge support to IMG activities rapidly as needed.

Equally important as this flexibility was the IMG’s ability to adapt to changing situations and to have all partners on the same page when doing so. This included procedural issues, such as updating the format of IMG monthly progress reports as the focus of the IMG’s work changed and merging or splitting subgroups when relevant and hibernating subgroups that had completed their tasks—for example, hibernating the finance group once budgeting was completed, or creating a working group on IPV supply as part of the implementation group to closely monitor and allocate the available vaccine.

The IMG’s flexibility was also seen when dealing with technical issues, such as IPV supply. While the IMG partners, particularly UNICEF’s Supply Division, worked hard to get accurate projections of vaccine availability, several consecutive reductions in IPV supply continued to haunt the program as demand continued to firm up. All partners agreed to use the risk-based tiering system to prioritize supply of IPV to those countries most in need. Associated decisions were made jointly and communicated in a single voice to affected countries, with a focus on moving forward in the most efficient and effective way whenever new situations or issues emerged.

The IMG used existing tools where possible, for example, with its approach to strengthen RI in the 10 focus countries [6]. Rather than introduce a new process, the IMG and its RI subgroup engaged in ongoing efforts to develop a single annual plan to guide immunization activities and provided catalytic funding for specific priority activities identified by each country, rather than for a preset activity defined by the IMG.

### Leveraging SAGE and the POB

The IMG had dual reporting lines to SAGE and to the POB through the Strategy Committee. The IMG benefitted from the engagement of these high-level bodies, from both the immunization and the polio sides. SAGE was responsible for the policies regarding IPV introduction and approving the timelines for the switch from tOPV to bOPV. Given that SAGE is the main policy-making body within the global immunization community, its endorsement of relevant objective 2–related policies was a critical step to enabling widespread country implementation under very tight timelines.

Because the POB is made up of the heads of each GPEI partner agency, it yielded significant influence. Providing regular updates to the POB and seeking its concurrence with the IMG’s proposed directions ensured high-level engagement and support within each organization. The IMG used its interactions with the POB to highlight areas of concern and seek POB interventions to unblock obstacles as needed. POB’s political advocacy and interventions were key factors in securing country-level commitment, and their support of the IMG’s financial requests, and the funds that came with that support, was essential for countries to meet the Endgame timelines.

### Proactive Communications

Providing regular, clear updates to all partners was a priority for the IMG and was seen as critical for ensuring broad engagement and commitment to the work on objective 2. Throughout the IMG’s work, regular communications (eg, information notes, job aids, training materials, and predeveloped PowerPoint presentations) were developed and disseminated for use by the communications subgroup. The IMG produced both scientific information packages as well as simple, clear documents that would be useful to frontline health care workers. These documents, which were translated into French, and often Spanish, Russian, and Arabic, allowed the IMG to keep regions, countries, and partners up to date as new information became available or situations changed. Early on, the IMG agreed to develop a Web site, hosted by WHO’s immunization program, where all information on objective 2 could be accessed publicly. This Web site was updated regularly and became a critical tool for the IMG (http://www.who.int/immunization/diseases/poliomyelitis/endgame_objective2/en/, accessed 7 October 2016).

In addition to producing materials, the IMG and regional offices also prioritized including sessions on IPV introduction and the tOPV-bOPV switch at key meetings of EPI managers, and when needed, organizing separate objective 2 focused meetings. These meetings ensured that countries’ senior immunization staff understood the rationale for objective 2 and had access to the latest information. IMG members from all partner agencies regularly attended a variety of meetings at regional and global levels to give updates and answer questions. The IMG, with the support of the Task Force for Global Health, also organized a series of Web-based seminars on key topics to reach partners and colleagues in an interactive, efficient, and widespread manner.

### Common Tools

With the support of the Task Force for Global Health, the IMG developed a detailed annual work plan, which was updated and reviewed monthly. This was a critical tool to ensuring that all critical activities were completed on time and that any potential delays were identified in advance, so that the IMG could explore ways to mitigate the delay and/or its impact. This common work plan allowed all partners and IMG members to monitor and get updates on IMG activities. The IMG also maintained a dedicated section of the WHO’s Immunization Repository that tracked country progress toward IPV introduction and the switch. Updated regularly by regions and headquarters staff, the repository provided a single resource from which all IMG partners could get up to date information on IPV introduction and OPV withdrawal in each country. A report was generated monthly and reviewed on the IMG call to reflect progress. While time consuming, these activities were critical to the IMG staying on track and flagging potential challenges and problems before they escalated.

## CONCLUSIONS

Ambitious goals were posed to the IMG in 2013. Although there were unexpected challenges, the success of the IMG’s work can be seen throughout the articles in this supplement. All 155 countries and territories that were using tOPV in 2015 reported that they had withdrawn it by May 2016 [7]. All 126 countries using only OPV in 2012 agreed to introduce IPV by the end of 2015, even though only 105 (83%) have actually introduced it owing to supply shortages. The work of the RI subgroup has accelerated identification of the impact and potential use of polio funded assets to strengthen RI.

The IMG’s success benefitted from the existing infrastructure built by GPEI. The IMG also benefited from EPI infrastructure that has been built up in a majority of countries worldwide since the 1970s. EPI programs have gained substantial experience in introducing new vaccines during the last 15 years, which enabled the introduction of IPV within short timelines. The IMG also benefitted from the high profile of polio eradication activities.

The level of commitment and dedication to polio at the national level—from the ministers to the health care workers themselves—meant that, once the resolutions were endorsed at the World Health Assembly and policies were set by SAGE, there was widespread uptake and rapid implementation. Finally, the IMG benefitted from the availability of the financial resources necessary to do its work, which were fundraised for and provided by GPEI.

As the IMG’s work slows down until the preparations for the withdrawal of all OPV can begin, the work of the IMG thus far can be used as a model for other coordinated global health initiatives beyond the polio sphere; these lessons learned highlight the importance and effectiveness of strong leadership, collaboration, adequate resources, and, most of all, dedication and commitment from immunization staff at the global, regional, and country levels.

## Notes

***Disclaimer.*** The findings, interpretations, and conclusions expressed in this article are those of the authors and do not necessarily reflect the policies or views of their respective organizations.

***Financial support.*** This work was supported by the Bill & Melinda Gates Foundation (grant OPP1095024 which included support for M. F.). Gavi, to support its role in the polio eradication efforts, received funds from the UK Department for International Development, the Norwegian Agency for Development Cooperation, and the Bill and Melinda Gates Foundation.

***Supplement sponsorship.*** This work is part of a supplement coordinated by the Task Force for Global Health with funding provided by The Bill and Melinda Gates Foundation and the Centers for Disease Control and Prevention.

***Potential conflicts of interest.*** M. F. was supported by a grant from the Bill & Melinda Gates Foundation. A small portion of the above-described funding received by Gavi was used to support Gavi Secretariat staff, including A. B. All other authors: no conflicts. All authors have submitted the ICMJE Form for Potential Conflicts of Interest. Conflicts that the editors consider relevant to the content of the manuscript have been disclosed.
